# A practical guide to the management of immune thrombocytopenia co-existing with acute coronary syndrome

**DOI:** 10.3389/fmed.2024.1348941

**Published:** 2024-04-11

**Authors:** Alaa Rahhal, Drew Provan, Waleed Ghanima, Tomás José González-López, Khaled Shunnar, Mostafa Najim, Ashraf Omer Ahmed, Waail Rozi, Abdulrahman Arabi, Mohamed Yassin

**Affiliations:** ^1^Pharmacy Department, Hamad Medical Corporation, Doha, Qatar; ^2^Barts and The London School of Medicine, Queen Mary University of London, London, United Kingdom; ^3^Østfold Hospital and Institute of Clinical Medicine, University of Oslo, Oslo, Norway; ^4^Department of Hematology, Hospital Universitario de Burgos, Burgos, Spain; ^5^Cardiology Department, Hamad Medical Corporation, Doha, Qatar; ^6^Internal Medicine Department, Rochester Regional Health—Unity Hospital, New York, NY, United States; ^7^Internal Medicine Department, Yale New Haven Health, Bridgeport, CT, United States; ^8^Hematology Department, National Centre for Cancer Care and Research, Hamad Medical Corporation, Doha, Qatar; ^9^College of Medicine, Qatar University, Doha, Qatar

**Keywords:** immune thrombocytopenia, acute coronary syndrome, corticosteroids, intravenous immunoglobulin, thrombopoietin receptor agonist, rituximab

## Abstract

**Introduction:**

Immune thrombocytopenia (ITP) management with co-existing acute coronary syndrome (ACS) remains challenging as it requires a clinically relevant balance between the risk and outcomes of thrombosis and the risk of bleeding. However, the literature evaluating the treatment approaches in this high-risk population is scarce.

**Methods and Results:**

In this review, we aimed to summarize the available literature on the safety of ITP first- and second-line therapies to provide a practical guide on the management of ITP co-existing with ACS. We recommend holding antithrombotic therapy, including antiplatelet agents and anticoagulation, in severe thrombocytopenia with a platelet count < 30 × 10^9^/L and using a single antiplatelet agent when the platelet count falls between 30 and 50 × 10^9^/L. We provide a stepwise approach according to platelet count and response to initial therapy, starting with corticosteroids, with or without intravenous immunoglobulin (IVIG) with a dose limit of 35 g, followed by thrombopoietin receptor agonists (TPO-RAs) to a target platelet count of 200 × 10^9^/L and then rituximab.

**Conclusion:**

Our review may serve as a practical guide for clinicians in the management of ITP co-existing with ACS.

## Introduction

Acute coronary syndrome (ACS) presents as unstable angina (UA), acute non-ST-elevation myocardial infarction (NSTEMI), or acute ST-elevation myocardial infarction (STEMI), which are considered cardiac emergencies, requiring prompt interventions, including the initiation of antithrombotic therapy with coronary angioplasty. Dual antiplatelet therapy (DAPT), including aspirin and a P2Y12 receptor inhibitor, is considered the cornerstone of ACS management as per the international clinical practice guidelines, including the American College of Cardiology Foundation (ACCF)/American Heart Association (AHA) and the European Society of Cardiology (ESC) (1, 2). Treatment with DAPT reduces the risk of both stent thrombosis and subsequent ischemic events; however, it increases the risk of bleeding (3, 4). Immune thrombocytopenia (ITP) is an acquired autoimmune disorder characterized by a low platelet count caused by platelet destruction along with impaired platelet synthesis. It is considered a rare hematological disorder with an estimated incidence in the general population of 2 to 5 per 100,000 persons (5).

The management of ITP co-existing with ACS is a challenging situation for healthcare providers, as this population is at a higher risk of bleeding and thrombosis (6). To minimize the risk of bleeding among patients with thrombocytopenia and co-existing ACS, McCarthy et al. proposed performing percutaneous coronary intervention (PCI) through radial access if the platelet count >50 × 10^9^/L without active bleeding and using drug-eluting stent (DES) instead of a bare-metal stent (BMS) with minimizing DAPT duration to 1 month followed by clopidogrel monotherapy thereafter (7). For ITP management, the American Society of Hematology (ASH) clinical practice guidelines recommend initial pharmacological treatment with corticosteroids with or without intravenous immunoglobulin (IVIG) followed by second-line therapies, including thrombopoietin receptor agonists (TPO-RAs), rituximab, or splenectomy for non-responders or those dependent on corticosteroids with platelet counts < 30 × 10^9^/L (5).

Nevertheless, corticosteroids are associated with an increased bleeding risk if used with DAPT and may worsen myocardial healing in ACS, and the remaining second-line ITP agents are associated with an increased risk of thrombosis (5, 8). Such considerations complicate the management of ITP co-existing with ACS. To the best of our knowledge, there are no current guideline recommendations or consensus reports to guide clinicians on the management of this high-risk cohort. In this review, we examined the evidence to date and provided our opinion on future directions and management strategies for ITP co-existing with ACS.

## Search strategy

We searched PubMed and Embase databases for the studies published in English exploring the management of ITP co-existing with ACS. We used the following terms: “Immune Thrombocytopenia Purpura,” “Immune Thrombocytopenia,” “Acute Coronary Syndrome,” “Percutaneous Coronary Intervention,” “Coronary Artery Bypass Graft,” “corticosteroids,” “Intravenous immunoglobulin, “Thrombopoietin receptor agonists,” “eltrombopag,” “avatrombopag,” “romiplostim,” and “Rituximab.” “AND” and “OR” were used as Boolean operators to combine the terms. The literature search included all articles published until 5 October 2022. The reference lists of the retrieved articles were manually screened.

## ITP pharmacological therapy

### Corticosteroids

Corticosteroids are the recommended first-line treatment for newly diagnosed ITP in adult patients who require therapy, i.e., platelet count < 30 × 10^9^/L or any platelet count with associated bleeding. In patients with a platelet count ≥ 30 × 10^9^/L who require antiplatelet or anticoagulation therapy, corticosteroids may be considered (5). Corticosteroids are widely available at a low cost but are associated with significant multi-system side effects. Specifically, corticosteroids can precipitate or exacerbate classical risk factors of coronary artery disease (CAD), such as hypertension, impaired glucose tolerance, and hypercholesterolemia (5).

The ASH clinical practice guidelines for ITP management do not give preference to prednisolone over dexamethasone but highlight that platelet recovery at 7 days may be faster and more sustained with dexamethasone (5). A recent meta-analysis of randomized controlled trials by Xiao and colleagues supported this notion. Patients with newly diagnosed primary ITP who received high-dose dexamethasone had a significantly higher overall response than standard-dose prednisone. This was not associated with a significantly different incidence of side effects, including arthralgia, elevated blood pressure, hyperglycemia, or mood disorders (9).

ACS is a proinflammatory, prothrombotic state. Corticosteroids have long been hypothesized to be beneficial for patients with acute myocardial infarction (AMI) (10). However, concerns for corticosteroid effects on wound healing, myocardial wall thinning, and potential myocardial rupture make them unfavorable agents in the setting of ACS. Moreover, acute and chronic corticosteroid use has been reported to increase the risk of myocardial infarction (MI), not necessarily in the presence of conventional risk factors for CAD (11). Proposed mechanisms include an increase in clotting factor production and inducing coronary vasospasm (12–14). Interestingly, it has been shown that corticosteroids may result in a 26% mortality benefit in AMI without a clear association with myocardial rupture (8). Notably, many of the studies included in this analysis were before the advent of the current standard medical treatment in ACS and, more importantly, before the widespread availability of PCI.

The comparative safety of different corticosteroids in patients with concomitant newly diagnosed ITP and ACS is based on observational data. Larger doses (i.e., a daily dose of prednisolone equivalent to more than 10 mg), as well as longer duration of therapy, especially in the first 30 days of use, may confer a higher risk (15, 16). Hence, it can be inferred that prednisone may be safer than dexamethasone in this population, as it is used at a lower dose. Nevertheless, an important caveat is that patients with ACS require DAPT and anticoagulation, increasing the risk of bleeding in the setting of ITP. Consequently, faster platelet recovery is a priority, and this may be better achieved with dexamethasone (9). Each corticosteroid carries an important advantage; dexamethasone helps achieve faster platelet recovery, facilitating earlier use of antithrombotic therapy for ACS but might increase the risk of MI as larger doses are needed, while prednisolone might be a safer option but could result in late platelet recovery, which could delay antithrombotic therapy for ACS. Therefore, the treating clinician may choose the agent that best aligns with the patient’s profile, considering the risk stratification of ACS, the urgency of coronary angiography, and PCI, bleeding, and thromboembolic risks.

The standard high-dose dexamethasone regimen to treat ITP is 40 mg per day for 4 days. Prednisone is given at a dose of 0.5–2 mg/kg daily for 2 weeks followed by a tapering regimen (5, 17). Current evidence suggests that prednisolone equivalent doses as low as 7.5 mg daily were found to increase the risk of cardiovascular complications including AMI (15, 16). In the setting of ITP with ACS, we recommend using the lowest effective dose of prednisolone or a short course of high-dose dexamethasone under close monitoring for platelet response and the occurrence of new thromboembolic events.

### Intravenous immunoglobulin

IVIG is considered one of the first lines of managing adults diagnosed with ITP. It is usually given if a faster platelet recovery is required, in cases of poor response to corticosteroids, concurrent contraindications to steroids, in the presence of active bleeding, or a high risk of bleeding (17, 18). It has been demonstrated that the concurrent use of corticosteroids and IVIG results in a shorter duration of complete remission and an overall response, without significant difference in adverse reactions (19, 20). In ITP, it can be administered at an initial dose of 1 g/kg as a one-time dose or 0.4 g/kg per day for 5 days and might be repeated if the response is suboptimal (17). IVIG has been shown to increase the likelihood of venous and arterial thromboembolic events (TEE). The first association of IVIG administration with thrombotic events was reported in 1986 when two patients had MI and two patients had a stroke after the infusion (21). The incidence of IVIG-induced thrombosis is estimated to be 1–16.9% as demonstrated in two retrospective studies with MI and stroke as the predominant arterial thrombotic events (22, 23). Cardiovascular events following immunoglobulin therapy have always been a challenge as the medical conditions managed with IVIG may contribute to ACS. Consequently, in 2013, the FDA mandated that a black box warning of increased risk of thrombosis be included on IVIG products (24).

We have identified a total of 16 cases of IVIG-induced MI as demonstrated in Table 1 (25–37). Certain risk factors were found to increase the risk of thrombosis with the use of IVIG infusion, including previous history of atherosclerotic diseases, thrombosis, concurrent hypercoagulable status, age of more than 45 years, and an IVIG daily dose of more than 35 g (38). Moreover, patients with ITP were found to have a higher incidence of thrombosis upon receiving IVIG than other pathological conditions treated with IVIG (38, 39). Taking all of the previous information into consideration, in the setting of ITP with ACS, we recommend using IVIG with corticosteroids among patients with profound thrombocytopenia, e.g., platelet count < 30 × 10^9^/L, or refractory thrombocytopenia despite corticosteroids, with a daily dose capping of 35 g (e.g., 0.5 g/Kg).

**Table 1:** Case reports of IVIG-induced myocardial infarction.

Author, Year	Age and sex	IVIG dose	Indication	Cardiac risk factors	Type and time to MI (From 1st dose of IVIG)	Outcome
Tan, 2008	23, Female	60 g for 2 doses	Thrombocytopenia in the setting of concurrent SLE and aPL	None	STEMI (LAD), 14 days	Survival
Elkayam, 2000	60, Male	660 mg/kg/day infusion for 3 days	Relapsing polychondritis	Hypertension	NSTEMI (thrombolytic therapy), 10 days	Survival
	41, Female	1 g/kg/day infusion for 2 days every month for 12 months (uneventful) then 2nd cycle due to relapse	Anti-Jo1 positive polymyositis with progressive interstitial lung disease	FHx of CAD, steroid-related adverse effects after the 2nd cycle (marked obesity, hypertension, diabetes mellitus requiring insulin)	STEMI, 6 days after the 3rd dose of the 2nd cycle of IVIG	Survival
	67, Male	400 mg/kg/day for 5 days	Chronic inflammatory demyelinating polyneuropathy	Hypercholesterolemia	Non-Q wave MI (LAD), few hours after 1st infusion	Survival (IVIG treatment was renewed without further complication after PTCA treatment for the NSTEMI)
	67, Male	400 mg/kg/months for 5 days every month	Systemic castleman disease	None	Inferior MI, day 4 of the 5th IVIG course	Survival
Eliasberg, 2007	43, Male	400 mg/kg (one dose for thrombocytopenia prior to CAG)	Antiphospholipid syndrome with steroid-dependent thrombocytopenia	Obesity, a positive family history of CAD, and a smoking habit (50 pack years of cigarettes), anterior NSTEMI 1 week prior to IVIG (he did not receive antiplatelet or anticoagulant therapy but rather was treated with nitrates and beta blockers alone)	Anterior STEMI (MI reinfarction), 1 h after initiation of IVIG infusion	Survival (CAG was canceled due to thrombocytopenia of 29,000, no antiplatelet or anticoagulant)
Paolini, 2000	78, Female	400 mg/kg daily (30 g) for 5 days	ITP	Severe hypertension, angina	Anteroseptal MI, 1 day after completion	--
Stamboulis, 2004	39, Male	0.5 g/kg/day for 5 days	Chronic Inflammatory Demyelinating Polyneuropathy in Association with a Monoclonal Immunoglobulin G Paraprotein	Heavy smoker	MI (CAG no lesion, mild anterior dyskinesia), 6 weeks after IVIG	--
Barsheshet, 2007	72, Male	0.4 g/kg per day for 5 days (32 g)	GBS	hypertension, hypercholesterolemia, IHD with PTCA, and stent implantation to LAD on account of stable angina pectoris 9 years ago	STEMI (CABG), 3 h following the start of infusion	--
Vinod, 2014	69, Male	0.4 g/kg/day × 5 days	GBS	None	Anterior STEMI (thrombolytics), when the last dose of IVIG was just about to be completed	Survival
Stenton, 2005	81, Male	IVIG 0.5 g/kg daily (38 g)	toxic epidermal necrolysis secondary to allopurinol	hypertension, angina, hypercholesterolemia, type 2 diabetes, chronic renal failure	NSTEMI, 30 min following the start of the IVIG infusion	Survival
Davé, 2007	65, Male	treated for 6 years with monthly IVIG [Polygam] without complications. 400 mg/kg (40 g) [Gammagard] tried for the 1st time	CVID	History of CAD and CABG before the diagnosis of CVID, hypertension, hyperlipidemia, and diet-controlled type 2 diabetes Mellitus, chronic stable angina with exercise 1 week prior to the Gammagard infusion	NSTEMI (repeat CABG), toward the end of the infusion of Gammagard	Survival
Mizrahi, 2009	76, Female	IVIG 2 mg/kg every month	Myasthenia gravis	None	NSTEMI (refused CAG), 2 h after IVIG (first day of her 3rd cycle)	Survival
Vucic, 2004	80, Female	0.6 g mg/kg every month	CIDP	None	STEMI (RCA), 336 h (received 52 IVIG treatments before this event)	--
Hefer, 2004	82, Male	37.5 g of IVIG (day 1 of admission), total of 65 g received by day 2 before the event	CML with refractory ITP	Hypertension	STEMI (no CAG), 3 h after finishing the infusion of IVIg	Survival
Zaidan, 2003	47, Male	0.25 g/kg/day |q4hr| increased after day 2 to 0.4 g/kg/day	GBS	Smoker, familial hypercholesterolemia, STEMI 3 weeks earlier	Inferior STEMI, during the 3rd dose	Survival

### Thrombopoietin receptor agonists

Currently, there are five commercially available TPO-RAs, including eltrombopag, avatrombopag, lusutrombopag, romiplostim, and recombinant human thrombopoietin (rhTPO). Eltrombopag is an oral, small, non-peptide molecule that initiates thrombopoietin receptor signaling, thereby inducing cell proliferation, differentiation, and maturation in the megakaryocytic lineage (40). Avatrombopag is a small-molecule TPO-RA that mimics the biological effects of endogenous TPO on platelet production. It was approved by the US Food and Drug Administration (FDA) in 2018, for treating thrombocytopenic disorders including ITP and chronic liver disease-induced thrombocytopenia (41). Lusutrombopag is a chemically synthesized orally active small-molecule TPO-RA that activates the signal transduction pathway in the same manner as endogenous TPO, thereby upregulating platelet production. It was approved in Japan in 2015 for use in patients with thrombocytopenia and chronic liver disease who are undergoing invasive procedures, and it is FDA-approved for liver disease-associated thrombocytopenia but not yet approved for ITP (42). Romiplostim is a novel peptide molecule that stimulates the megakaryocytopoiesis and increases the platelet count in the same manner as TPO (43). RhTPO is a glycosylated TPO that was approved in China as a second-line option for ITP (44).

According to the latest ASH guidelines for ITP management, the first-line therapy for newly diagnosed ITP is a short course of corticosteroids. For individuals with ITP ≥3 months who depend on corticosteroids or respond poorly to corticosteroids, the ASH guidelines suggest using second-line therapies, including TPO-RAs (once-daily oral eltrombopag or once-weekly subcutaneous injection romiplostim), rituximab, or splenectomy after appropriate immunizations (5). A recently published meta-analysis of 20 randomized controlled trials comprising 2,207 patients with ITP demonstrated that avatrombopag, lusutrombopag, eltrombopag, and romiplostim demonstrated a significantly better platelet response defined as platelet counts ≥ 30 or 50 × 10^9^/L during the treatment period compared with placebo (OR 36.90, 95%CI 13.33–102.16; OR 19.33, 95%CI 8.42–44.40; OR 11.92, 95%CI 7.43–19.14; OR 3.71, 95%CI 1.27–10.86, respectively) (45).

Because of the higher incidence of thrombosis in patients with ITP than in the healthy population, it was recognized as a unique complication of ITP (46). However, the pathogenic mechanisms responsible for the increased thrombotic risk associated with TPO-RAs have not yet been identified (47). The excessive increase in platelet count among patients treated with TPO-RAs, and the production of immature, more active platelets may partially explain the reason for high risk of thrombosis (48). Interestingly, an excessive increase in platelet count to 200 × 10^9^/L was associated with an increased risk of thrombosis within a median time of 21.5 days (range, 15 to 53) from the first dose of eltrombopag in a randomized controlled trial of eltrombopag use (49). In a meta-analysis of 2,207 patients receiving TPO-RAs for ITP, there were no significant differences between the TPO-RAs and placebo in terms of thrombosis (45). However, using surface under the cumulative ranking curve (SUCRA), with a larger SUCRA indicating a higher incidence of the outcome, the combination of rhTPO and rituximab had the highest SUCRA value for thrombosis of 74.3, followed by rituximab of 71.7 alone, and then the remaining TPO-RAs (45).

As demonstrated in Table 2 of studies evaluating the efficacy and safety of TPO-RAs, there was no dose-dependent thrombotic risk with TPO-RA use. Additionally, arterial thrombosis in the form of ACS was rare (49–63). Thus, TPO-RAs for ITP in the setting of ACS might be used at the regular dosing regimens for ITP. Nevertheless, eltrombopag undergoes extensive hepatic metabolism, and thus, its use with a high-intensity statin (atorvastatin and rosuvastatin) alters the elimination of statin therapy through the inhibition of OATP1B1 transporters, requiring lower doses of statin and frequent monitoring for statin-induced hepatotoxicity and myopathy (64). Therefore, eltrombopag might be the least favorable oral TPO-RA in ACS.

**Table 2:** Literature evaluating the safety of TPO-RA use.

Study ID	Intervention and control	TPO-RA dose	Duration	Thrombotic events	Time to thrombosis	PLT count at time of thrombosis
Afdhal, 2012	Eltrombopag vs. placebo	Eltrombopag 75 mg	14 days	TPO-RA: 6 PVT^§^ Placebo: 1 MI, 1 PVT	TPO-RA: 1–38 days	TPO-RA: 33–417 × 10^9^/L
					Placebo: 20–128 days	Placebo: 83 × 10^9^/L
Hidaka, 2019	Lusutrombopag vs. placebo	Lusutrombopag 3 mg daily	7 days	TPO-RA: 1 PVT	TPO-RA: 14 days	TPO-RA: 70 × 10^9^/L
				Placebo: 1 superior mesenteric vein thrombosis	Placebo: 20 days	Placebo: 60 × 10^9^/L
Tateishi, 2019	Lusutrombopag vs. placebo	Lusutrombopag 2 mg, 3 mg, 4 mg	7 days	Lusutrombopag 2 mg: 1 PVT	-	TPO-RA: 37–91 × 10^9^/L
				Lusutrombopag 3 mg: none		
				Lusutrombopag 4 mg: 2 VTE (1 PVT, mesenteric vein thrombosis)		
				Placebo: 1 mesenteric vein thrombosis		Placebo: -
Peck-Radosavljevic, 2019	Lusutrombopag vs. placebo	Lusutrombopag 3 mg daily	7 days	TPO-RA: 2 (1 left intrahepatic artery thrombosis, 1 left ventricular thrombus^*^)	-	TPO-RA: 62 and 119 × 10^9^/L
				Placebo: 2 splanchnic thrombosis		Placebo: -
Terrault, 2018	Avatrombopag vs. placebo	Avatrombopag 60 mg ➔ PLT < 40	5 days	TPO-RA: 1 PVT	TPO-RA: 18 days	TPO-RA: 61 × 10^9^/L
		Avatrombopag 40 mg ➔ PLT < 50		Placebo: 2 (1 MI, 1 PE)	Placebo: -	Placebo: -
Jurczak, 2018	Avatrombopag vs. placebo	Avatrombopag 5-40 mg	6 months	TPO-RA: 4 (1 DVT, 1 PE, 1 CVA, 1 jugular vein thrombosis)	TPO-RA: 8–335 days	TPO-RA: 39–271 × 10^9^/L
				Placebo: 0		
Kuter, 2018	Avatrombopag vs. placebo	Avatrombopag up to 100 mg daily	14 days	TPO-RA: 0	NA	NA
				Placebo: 0		
Bussel, 2014	Avatrombopag vs. placebo	Avatrombopag 2.5-20 mg	28 days	TPO-RA: 5 (1 DVT, 1 MI^**^, 1 retinal artery occlusion, 1 superficial thrombophlebitis, 1 stroke)	TPO-RA: -	TPO-RA: 19–571 × 10^9^/L
				Placebo: 0		
Cheng, 2011	Eltrombopag vs. placebo	Eltrombopag 50 mg	6 months	TPO-RA: 2 PE^***^, 1 DVT^***^	TPO-RA: 5–6 days	TPO-RA: 42–49 × 10^9^/L
				Placebo: 0		
Yang, 2016	Eltrombopag vs. placebo	Eltrombopag 25–75 mg	8 weeks	TPO-RA: 1 DVT	-	-
				Placebo: 0		
Bussel, 2009	Eltrombopag vs. placebo	Eltrombopag 50–75 mg	6 weeks	TPO-RA: 0	NA	NA
				Placebo: 0		
Tomiyama, 2012	Eltrombopag vs. placebo	Eltrombopag 12.5–50 mg	6 weeks	TPO-RA: 1 TIA	TPO-RA: 8 days	TPO-RA: 76 × 10^9^/L
				Placebo: 0		
Bussel, 2006	Romiplostim vs. placebo	Romiplostim 1, 3, or 6 μg/Kg SC weekly	6 weeks	TPO-RA: 0	-	-
				Placebo: 1 DVT		
Kuter, 2008	Romiplostim vs. placebo	Romiplostim 1–15 μg/Kg SC weekly	24 weeks	TPO-RA: 1 popliteal artery thrombosis, 1 CVA	TPO-RA: 147–224 days	TPO-RA: 11–107 × 10^9^/L
				Placebo: 1 PE		
Shirasugi, 2011	Romiplostim vs. placebo	Romiplostim 3 μg/Kg SC weekly	12 weeks	TPO-RA: 0	NA	NA
				Placebo: 0		

^*^Had history of CAD and LVT; ^**^Had significant CV history with CABG and TIAs; ^***^Had risk factors for thrombosis; ^§^five out of six events occurred when PLT > 200, and events after within a median of 8 days after last dose of therapy; PVT: portal vein thrombosis.

ITP management in the setting of ACS remains uncertain and challenging in view of the need for a balanced regimen between bleeding and thrombosis risk. Among patients with treatment-naive ITP and concurrent ACS who are either corticosteroid-dependent or corticosteroid-poor responders, we suggest using TPO-RA (avatrombopag, or once weekly subcutaneous injection romiplostim) as a second-line ITP therapy to target platelet count > 50 × 10^9^/L, permitting the use of DAPT, to a maximum platelet count of 200 × 10^9^/L to reduce the risk of TPO-RA-associated thrombosis. We recommend against using a combination therapy of TPO-RA and rituximab to reduce the risk of thrombosis.

### Rituximab

Rituximab is another frequently used second-line treatment modality in ITP. The mechanism of action responsible for its efficacy is not fully understood (65). Rituximab is an anti-CD20 monoclonal antibody that targets B cells. It was proposed that B-cell destruction will result in the underproduction of antibodies, hence the therapeutic benefits of ITP (66). However, more recent evidence showed that the rituximab effect is more complicated than we thought and it extends to involve the T cells. It was found that rituximab neutralizes the auto-reactive T cells and patients who responded to the therapy demonstrated normalization of the T-cell abnormalities (67–69). It is proposed that B cells might play a role in keeping the T cells active and targeting the T cells indirectly is the main drive behind the successful use of rituximab in ITP patients (65).

According to the most recent ASH guidelines for ITP management, rituximab is not the initial therapy of choice (5). However, it can be used as add-on therapy to corticosteroids if more emphasis is placed on achieving remission while accepting the potential side effects. Rituximab is one of the second-line options, in addition to TPO-RAs and splenectomy, in patients who are corticosteroid-dependent for 3 months or more or who showed no response to corticosteroids (5).

There are several case reports of the development of ACS, mostly STEMI, following rituximab infusion that was used for different medical conditions as shown in Table 3 (70–79). More than half the events occurred after the first dose of rituximab. Unfortunately, the exact doses of rituximab were not reported in most cases. It is worth mentioning that almost all reported cases of MI occurred during rituximab infusion or just a few hours afterward. There was only one reported case of delayed MI occurring within 24 h after the infusion and that is the only case in which the indication for rituximab was the treatment of ITP, which raises questions about whether the event was related to rituximab (77). To the best of our knowledge, there are no other reported cases of ACS in ITP patients following rituximab infusion. Zhou et al. compared rituximab plus recombinant human thrombopoietin (rhTPO) vs. rituximab alone for corticosteroid-resistant or relapsed ITP in a randomized controlled trial, and they found that only one patient died from MI out of the 77 participants in the rituximab plus rhTPO group (44). The patient was 77 years old with known cardiac risk factors and was labeled as a non-responder after 8 months of treatment. No deaths or cardiac events were reported in the rituximab monotherapy group. In another study, two out of 55 patients on rituximab developed venous thromboembolic events (VTE); one pulmonary embolism and one deep venous thrombosis; however, no cardiac events were recorded (80). A recent study in 2019 investigated the risk of thromboembolism of rituximab by looking into the adverse events reported from two randomized clinical trials (81). It was noted that the rate of VTE was higher in ITP patients treated with rituximab; however, the authors could not conclude whether these events were triggered by rituximab or caused by other confounding factors.

**Table 3:** Case reports of Rituximab-induced myocardial infarction.

Study	Age and sex	Rituximab dose	Indication	Cardiac risk factors	Type and time to MI	Outcome
Armitage, 2008	58, Male	First	CLL	Previous MI	Two have MI during the infusion (one of them was with a test dose of 25 mg) and the third at the completion of infusion	Survival
	61, Male		BL	+Risk factors		Survival
	72, Male		BLL	+Risk factors		Death
Arunprasath, 2011	60, Male	First	DLBCL	Diabetes	AWMI, 15 min after starting the infusion	Survival
Renard, 2013	52, Male	Third, 375 mg/m^2^	MG	None	IWMI, 10 h after the infusion	Survival
Gogia, 2014	65, Male	First	SLVL	None	IWMI, after 5 min of starting the infusion	Survival
Van Sijl, 2014	70, Female	First and second	RA	Previous history of MI	ALWMI	Survival
	76, Female	Second	RA	None	AWMI	Survival
Keswani, 2015	46, Male	Second	DLBCL	Smoking: 25 pack-years	IWMI, halfway through the infusion	Survival
Verma, 2016	62, Male	First	NHL	None	IWMI, after 5 min of starting the infusion	Survival
Mehrpooya, 2016	52, Female	First dose, 375 mg/m^2^	ITP	HTN, mild CAD	IWMI, 24 h	Survival
Arenja, 2016	36, Male	Not specified	PR3-ANCA-positive granulomatosis with polyangiitis	Smoking: 20 pack-years, HTN, hypercholesterolaemia	AWMI	Survival
Sharif, 2017	58, Male	Fifth dose of 1,000 mg	RA + Scleroderma	HTN, smoking: 30 pack-years	AWMI, during infusion	Survival

Among patients with ITP and concurrent ACS who are either corticosteroid-dependent or poor responders, we recommend using rituximab without combining it with TPO-RA due to the increased risk of MI.

## General approach to ITP management with co-existing ACS

### ITP with platelet count < 30 × 10^9^/L

**Figure 1:**
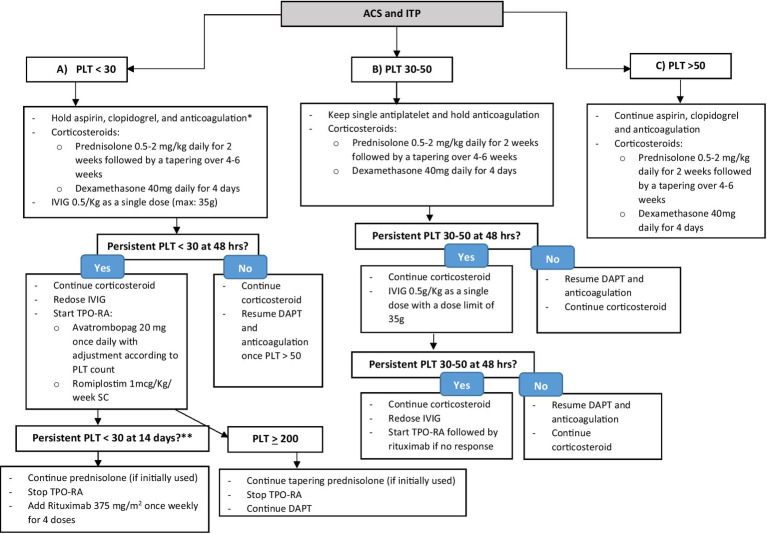
Stepwise approach of the management of ITP co-existing with ACS. ^*^After a multidisciplinary team evaluation of the case to assess risks and benefits. ^**^To consider fostamatinib in refractory ITP despite TPO-RA and rituximab.

The management of patients with severe thrombocytopenia in the setting of ITP with concurrent ACS is challenging and requires an individualized approach based on the anticipated short- and long-term prognosis of the thrombotic event in case of delayed intervention vs. the risk of bleeding resulting from antithrombotic therapy, taking into consideration patient’s age, refractoriness of ITP, and concurrent comorbidities. The evaluation of such a patient requires a multidisciplinary team approach. We advise holding antithrombotic therapy, including DAPT and anticoagulation, until platelet count is >30–50 × 10^9^/L after evaluating the risks and benefits in a multidisciplinary team to individualize the management, along with starting the first-line ITP treatment with corticosteroids, either low-dose prednisolone or short course of high-dose dexamethasone plus IVIG with dose limit of 35 g (e.g., 0.5 g/Kg) daily, as demonstrated in Figure 1. In case of an increase in platelet count within 48 h of initial treatment, we advise continuing corticosteroids; prednisolone with a tapering schedule over 4–6 weeks or dexamethasone for a total of 4 days, and resuming antithrombotic therapy once platelet count > 50 × 10^9^/L. Among P2Y12 inhibitors, we prefer clopidogrel over ticagrelor and prasugrel in view of its lower risk of bleeding (82, 83). In case of persistent platelet count < 30 × 10^9^/L within 48 h of initial therapy, in addition to corticosteroids, we recommend re-dosing IVIG with dose capping of 35 g along with starting a TPO-RA, including avatrombopag or romiplostim to a target platelet count of 200 × 10^9^/L. We advise against using eltrombopag in the setting of ACS in view of drug–drug interaction with high-intensity statin therapy that is recommended in ACS, warranting dose reduction of statin therapy and close monitoring of liver enzymes and myopathy (64). In case of persistently severe thrombocytopenia within 14 days of TPO-RA, switching to TPO-RA is recommended. Rituximab 375 mg/m^2^ once weekly for four doses might be added. We advise against combining TPO-RA and rituximab therapy in view of the increased risk of thrombotic events (44, 45). In case of refractory thrombocytopenia despite IVIG, corticosteroids, TPO-RAs, and rituximab, the use of fostamatinib, which is a tyrosine kinase inhibitor recently approved in 2018 by the FDA for the treatment of chronic ITP unresponsive to previous therapies, might be considered (84). However, the thrombotic risk of fostamatinib has not yet been well evaluated (85). In cases of active bleeding, platelet transfusion can be considered, yet its role in ITP remains controversial (86).

### ITP with platelet count 30–50 × 10^9^/L

Among patients with a platelet count of 30–50 × 10^9^/L due to ITP co-existing with ACS, we advise considering only a single antiplatelet, either aspirin or clopidogrel, holding anticoagulation for ACS, and starting prednisolone or dexamethasone for ITP, as shown in Figure 1. Within 48 h of therapy initiation, we advise to resume DAPT and parenteral anticoagulation, preferably a short-acting agent (i.e., unfractionated heparin) (87) in case of platelet count improvement to >50 × 10^9^/L. In case of persistent thrombocytopenia with platelet count of 30–50 × 10^9^/L, we recommend starting IVIG with a dose capping of 35 g while continuing the initial corticosteroid regimen. In the next 48 h, in case of no improvement in platelet count, we advise re-dosing IVIG and starting a TPO-RA, followed by rituximab if there is no response, as demonstrated in Figure 1.

### ITP with a platelet count of >50 × 10^9^/L

In the least severe form of ITP with a platelet count of >50 × 10^9^/L with co-existing ACS, we recommend continuing all antithrombotic therapies for ACS, including DAPT and parenteral anticoagulation, and starting corticosteroids for ITP as shown in Figure 1.

## Conclusion

ITP management with co-existing ACS is a growing dilemma as a clinically relevant balance between thrombosis and risk of bleeding needs to be achieved, especially since corticosteroids, the cornerstone therapy in ITP, might increase the risk of bleeding once combined with antithrombotic therapy in ACS, and the second-line agents in ITP might increase the risk of venous and arterial thrombosis. The literature evaluating the treatment approaches and outcomes in this high-risk population is scarce. Therefore, in this review, we attempted to summarize the available evidence on the safety of ITP therapies and provide a practical guide on the management of ITP co-existing with ACS. In general, we advise holding antithrombotic therapy in cases of severe thrombocytopenia with a platelet count < 30 × 10^9^/L after evaluating the risks and benefits in a multidisciplinary team, and then using a single antiplatelet agent if the platelet count falls between 30 and 50 × 10^9^/L. DAPT along with anticoagulation should be continued if the platelet count is >50 × 10^9^/L. We provide a stepwise approach to the management of ITP according to platelet count and response to initial therapy, starting with corticosteroids plus-minus IVIG with dosing capping. This can be followed by TPO-RAs to achieve a target platelet count of 200 × 10^9^/L. Finally, rituximab without combining it with TPO-RA to reduce the risk of thrombosis can be considered. Future studies are needed to evaluate the safety and effectiveness of the stepwise approach in the treatment of ITP co-existing with ACS.

## Author contributions

AR: Conceptualization, Data curation, Methodology, Resources, Software, Supervision, Validation, Visualization, Writing – original draft, Writing – review & editing. DP: Conceptualization, Methodology, Validation, Writing – review & editing. WG: Conceptualization, Validation, Writing – review & editing. TJG-L: Conceptualization, Validation, Writing – review & editing. KS: Data curation, Writing – original draft. MN: Data curation, Writing – original draft. AAh: Data curation, Writing – original draft. WR: Data curation, Writing – original draft. AAr: Conceptualization, Resources, Validation, Writing – review & editing. MY: Conceptualization, Funding acquisition, Methodology, Resources, Supervision, Validation, Writing – review & editing.
